# Adrenal Virilism

**Published:** 1914-11

**Authors:** 


**Adrenal Virilism.** Tuffier, Bull. de l'Acad. de Med., May 26, 1914, describes a case in a woman aged 62, with menorrhagia and glycosuria, relieved by diet, etc., to permit operation. This disclosed a fibro-lipomatous mass half the size of the kidney, involving each adrenal, atrophic left ovary, right ovary with a tumor the size of a large nut. The face was bearded and masculine in features; voice masculine; slight exopthalmos, almost confluent yellow spots on fore arms and hands, great muscular development; clitoris 4 c. m. long and covered by enlarged prepuce. Reference is also made to three cases in young girls reported by Gallais, Soc. de Psych. de Paris, March 21, 1912, and to other cases. There may be precocious development with great muscular strength at the age of 3 or 4. Ultimately, while the hypertrichosis persists, the strength fails, both physical and mental.

